# Methodology to simulate unsaturated zone hydrology in Storm Water Management Model (SWMM) for green infrastructure design and evaluation

**DOI:** 10.1371/journal.pone.0235528

**Published:** 2020-07-06

**Authors:** Min-cheng Tu, Bridget Wadzuk, Robert Traver

**Affiliations:** 1 Department of Civil Engineering, National Taipei University of Technology, Taipei, Taiwan; 2 Department of Civil and Environmental Engineering, Villanova University, Villanova, Pennsylvania, United States of America; Hellenic Agricultural Organization - Demeter, GREECE

## Abstract

Hydrologic models such as the USEPA Stormwater Management Model (SWMM) are commonly used to assess the design and performance of green infrastructure (GI). To accurately represent GI performance models used in design need to be able to address both the hydrology/hydraulics of the catchment and the GI unsaturated (vadose) zone hydrology. While hydrologic models, such as SWMM, address the need for catchment hydrology/hydraulics, they often simplify the unsaturated zone hydrology. This paper presents a methodology utilizing existing components of SWMM to represent unsaturated zone hydrology in an accessible format that does not require adjustments to the SWMM source code. The methodology simulated the unsaturated soil water movement by considering flow caused by differences of soil matric head and flow caused by gravity between soil layers with finite depth/length. The flow flux related to the soil matric head is a function of soil water diffusivity (D) and the soil moisture gradient, where D can be represented by a pump curve in SWMM. The flow flux related to gravity was controlled by unsaturated hydraulic conductivity (K) only and was also simulated by a pump. The methodology was compared to another variably saturated model, HYDRUS, with theoretical soils (with single layers of sand, loam, silt, and clay, as well as dual-layer scenarios). Field data was used to compare the methodology to HYDRUS and the SWMM LID (Low Impact Development) module. In all comparisons the presented methodology and HYDRUS delivered similar results for the vadose zone response to a storm event, while the LID module of SWMM exhibited slower water movement. The results showed that under natural conditions, the approximation of the presented methodology yielded satisfactory results to simulate flow through the unsaturated vadose zone.

## 1. Introduction

Green Infrastructure (GI) is an effective tool to mitigate urban nonpoint source pollution and restore the natural water cycle, which incorporates media and plants that utilize all aspects of the hydrologic cycle and plant transpiration to maximize water removal. There are other benefits of GI including mitigating water quality, promoting soil infiltration, promoting human health, promoting ecosystem services, mitigating air quality, and mitigating the urban heat island effect [1–7]. Recognizing the benefits of GI, many cities have started to adopt various types of GI at the city-wide scale [8–9], however, there remains a need for models that represent the vadose zone hydraulics to aid in design and understanding of system behavior. For example, water uptake by plant roots is an important part of GI function [10–12] and can depend on both soil moisture [13–14] and depth-dependent root density [15]. To accurately simulate soil moisture in the unsaturated (vadose) zone by the Richards equation [16–18] and Van Genuchten relation [19] during GI design and/or evaluation is the first challenge.

A second challenge is that due to the nature of nonpoint source control, strategically placing GI on a large, watershed scale is crucial. The model of choice should simulate watershed hydrology accurately. The final challenge, which aligns with the future trend of stormwater management, is that the model should be compatible with the emerging trend of smart real-time control stormwater systems [20].

**Fig 1:**
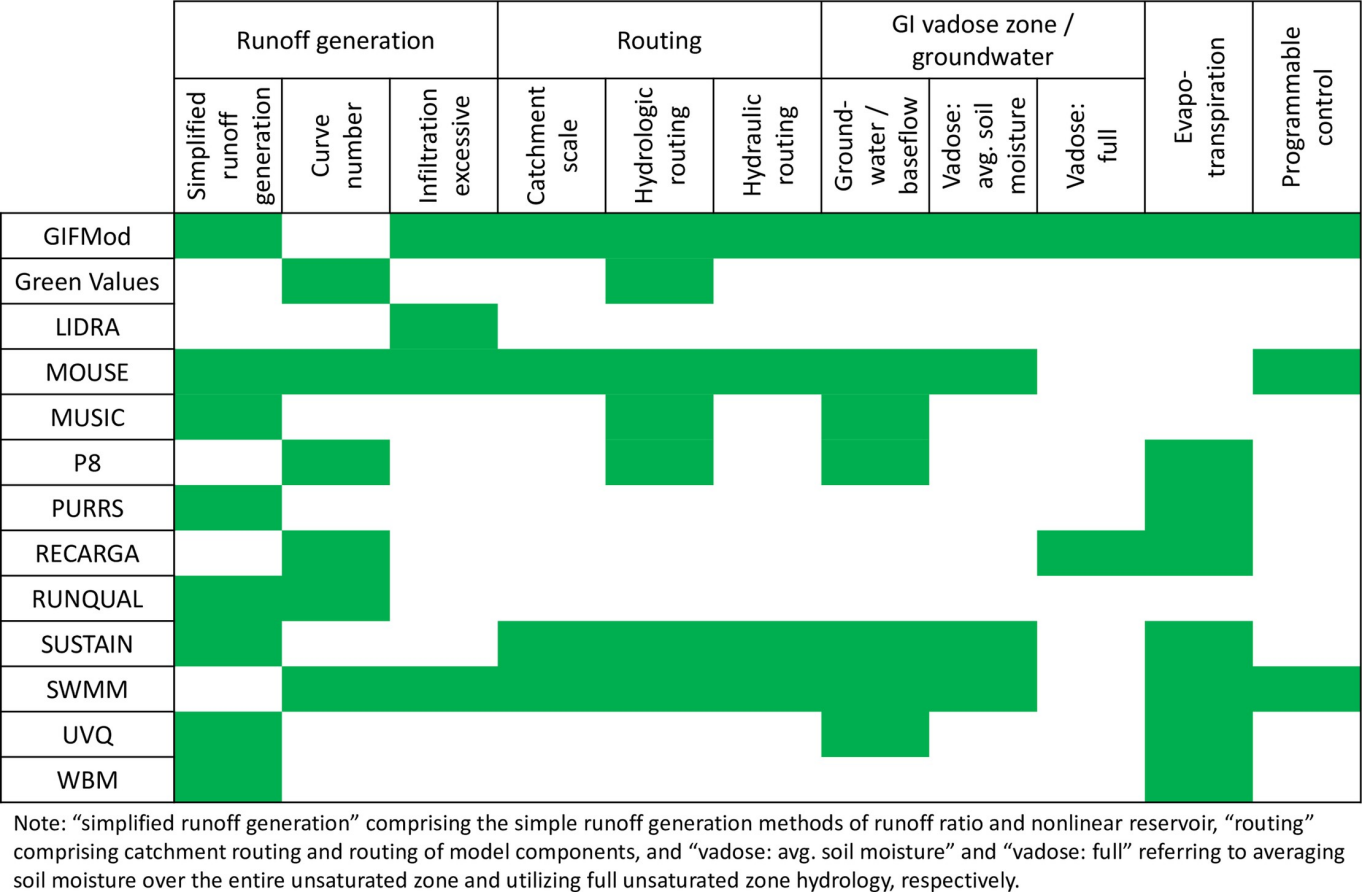
Comparison of models capable of simulating GI (green shading indicates capability).

There are many models that can simulate GI [21–23], as detailed in Fig 1. Several models can simulate hydrologic routing in the component scale, but not in the catchment scale (such as Green Values [24], MUSIC [25], and P8 [26]). Also, the capability of programmable control is not the same for all models. GIFMod [23] contains only the PID (Proportional–Integral–Derivative) controller. Whereas MOUSE [27] and SWMM [28] allow users to provide detailed control rules, which is helpful in simulating smart real-time control stormwater systems. As a result, GIFMod, MOUSE, and SWMM are the three models closest to the general needs of the stormwater design community. Although MOUSE is a popular model, it is proprietary and it has limited applications in the literature. GIFMod appears to have great potential but it is a new model introduced to users in 2017 so it is not widely used yet. SWMM is free software in the public domain and supported by a strong group of users and the United States Environmental Protection Agency (USEPA). The only key function missing from SWMM is the capability to simulate unsaturated zone hydrology; therefore, finding a solution to this drawback in SWMM will deliver a model that meets all users’ needs.

Only a few studies that simulate unsaturated zone hydrology in SWMM were found. Chen and Goldscheider [29] used SWMM to simulate a karst system with an unsaturated zone, but the unsaturated zone was considered as a lumped system. Their approach was similar to that of the existing groundwater and LID (Low Impact Development) modules of SWMM, which average the soil moisture in the whole unsaturated zone [28, 30–31]. Alternatively, Hudepohl et al. [32] circumvented the problem by coupling SWMM externally to HYDRUS [33], an unsaturated zone model adopting the Richards equation and complete Van Genuchten relation. It allowed SWMM users to simulate unsaturated zone hydrology, but such coupling required substantial modeling skill and was not accessible by most users. Lynn et al. [34] utilized HYDRUS as a tool to calibrate the relation between drainage flow and soil moisture in SWMM, which yields merely an approximation of the unsaturated zone hydrology. Moreover, it was feasible in only one soil texture (i.e., water not flowing through multiple soils with different properties) and for downward infiltration only. The only soil texture constraint is a limitation in GI application as there is usually more than one layer of soil, including the engineered soil and native soil.

There was no apparent methodology available to accurately simulate the unsaturated zone hydrology in SWMM without reprogramming the source code or coupling to external models. Therefore, an easier way is needed to include the unsaturated zone hydrology in SWMM. It was hypothesized that the mechanism of unsaturated zone hydrology can be simulated with high accuracy by using existing components of SWMM. The goal of this study was to provide a methodology to simulate unsaturated zone hydrology without modifying the source code of SWMM or relying on an external unsaturated zone model (e.g., HYDRUS) that may require more sophisticated data migration and modeler skill. For the rest of this paper, Section 2 (methodology) describes the proposed methodology, Section 3 (results) shows the application results of the methodology, and Section 4 (discussion) provided analyses for the application results and validation for the methodology. An application guide and an assistant EXCEL tool are available in the S1 Data and S1 File accompanying this paper.

## 2. Methodology

### 2.1. Simplifying the Richards equation to be used in SWMM

Water movement in the unsaturated soil can be described by the Richards Equation rewritten for soil water diffusivity *D* [35] (in [L^2^/T]) as shown by Eq 1:

$$\frac{\partial \theta }{\partial t}=\frac{\partial }{\partial x}\left[D\frac{\partial \theta }{\partial x}\right]+\frac{\partial }{\partial y}\left[D\frac{\partial \theta }{\partial y}\right]+\frac{\partial }{\partial z}\left[D\frac{\partial \theta }{\partial z}+K\right]-S_{w}$$ (1)

In Eq 1, $D=K\frac{\partial h}{\partial \theta }$ where *K* (in [L/T]) is the unsaturated hydraulic conductivity, *θ* is the volumetric soil moisture (unitless), and *h* is the soil water matric head (in [L]). In addition, *t* is time, *x* and *y* represents perpendicular and horizontal directions in the Cartesian coordinate system, *z* represents the vertical direction, and *S*_*w*_ is the sink term (in [1/T]) representing water removal rate from the soil by mechanisms such as root uptake or evaporation. Eq 1 assumes no anisotropy for the unsaturated hydraulic conductivity.

Assuming the soil moisture gradient exists in only the horizontal direction (i.e. $\frac{\Delta \theta }{\Delta x}$) and there is no water sink (such as roots), the 1-D horizontal water movement is:

$$I_{x}=D\frac{\Delta \theta }{\Delta x}$$ (2)

Similarly, the water movement in the vertical direction (assuming no soil moisture gradient in the horizontal directions) is:

$$I_{z}=D\frac{\Delta \theta }{\Delta z}+K$$ (3)

where *I*_*x*_ and *I*_*z*_ are flow flux [L/T] in the horizontal and vertical directions, respectively.

Eqs 2 and 3 are in a simple form that can be implemented in SWMM, which is delineated in Sections 2.2 and 2.3. The unsaturated hydraulic conductivity *K* in Eq 3 can be represented as a function of volumetric soil moisture [19]:

$$K(\theta )={K_{s}\Theta }^{0.5}{\left[1-{\left(1-\Theta^{1/m}\right)}^{m}\right]}^{2}$$ (4)

where $\Theta =\frac{\theta -\theta_{r}}{\theta_{s}-\theta_{r}}$ with *θ* the volumetric soil moisture, *θ*_*r*_ the residual volumetric soil moisture, and *θ*_*s*_ the saturated volumetric soil moisture. In Eq 4, *K*_*s*_ is the saturated hydraulic conductivity and $m=1-\frac{1}{n}$ where *n* is an empirical parameter for the water retention curve (also called SWCC: Soil Water Characteristic Curve).

Van Genuchten [19] also provided a presentation of soil water diffusivity as Eq 5 shows:

$$D(\theta )=\frac{(1-m)K_{s}}{\alpha m(\theta_{s}-\theta_{r})}\Theta^{\left(0.5-\frac{1}{m}\right)}\left({\left(1-\Theta^{\frac{1}{m}}\right)}^{-m}+{\left(1-\Theta^{\frac{1}{m}}\right)}^{m}-2\right)$$ (5)

where *α* is another empirical parameter for the water retention curve. All other parameters were identically defined as in Eq 4. Only the single-porosity Van Genuchten relation was considered.

### 2.2. Procedure to simulate the Richards equations for soil with a uniform texture

**Fig 2:**
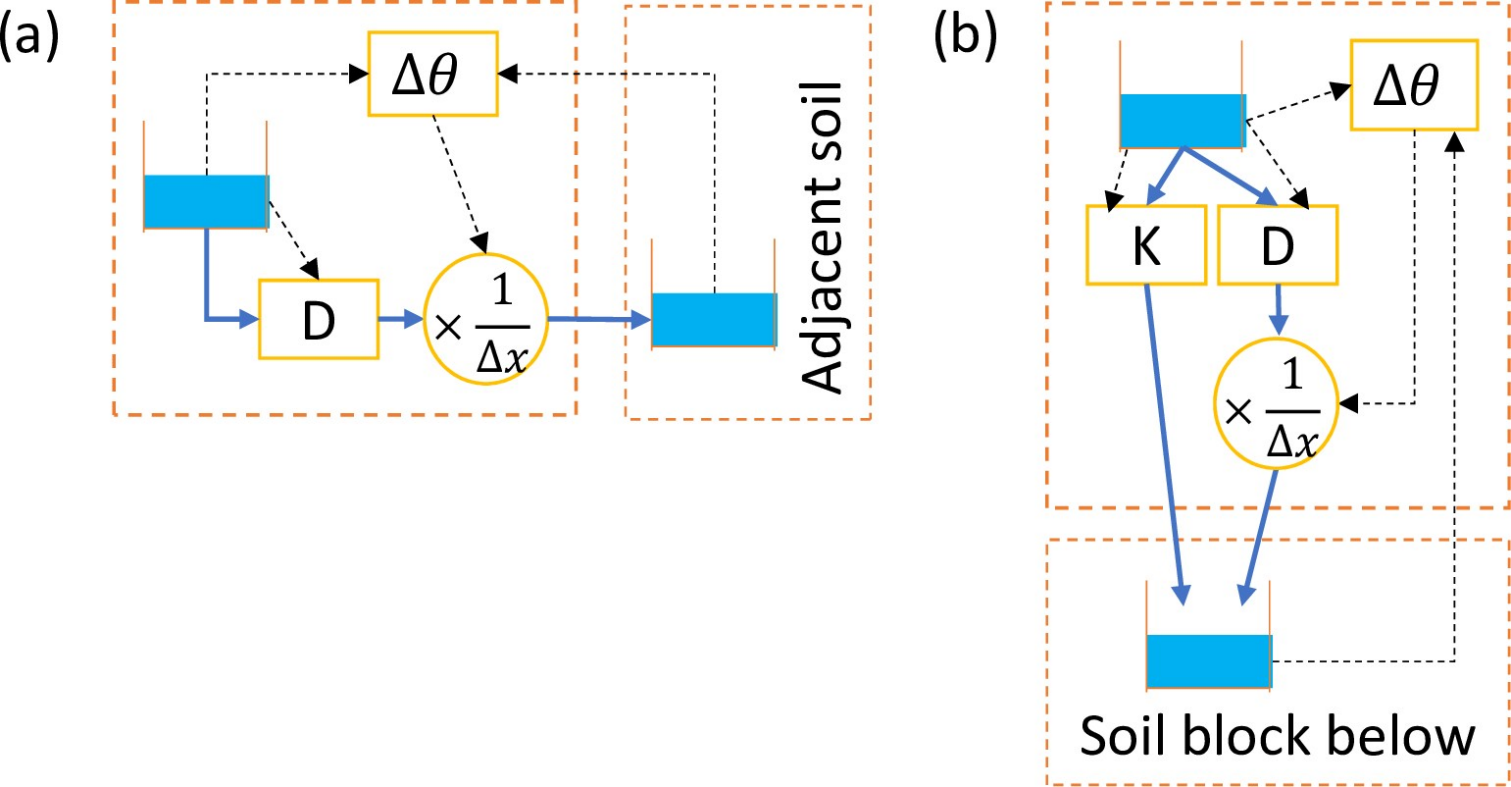
Basic mechanisms of the proposed methodology. Blue solid arrows indicate water movement, and black dashed arrows are flows of information. Blue water in storage units represents the amount of water in the soil block, so the volumetric soil moisture is represented by the height of the blue water divided by the total height of the storage unit.

The methodology considered the soil as multiple inter-connected blocks with properties (including soil moisture) homogeneous within a soil block. Dividing the modeled domain into multiple connected blocks is intuitive and was widely used by many models, such as GIFMod [23]. The basic mechanism of the proposed methodology is illustrated in Fig 2.

Fig 2(A) illustrates how horizontal unsaturated water movement was calculated. Component D, which is simulated by a Type 4 pump (flow rate decided by the water depth at the beginning node of the pump) in SWMM [28], pumps water from the storage according to a pump curve delineating the relationship between soil moisture and water diffusivity *D* (Eq 5). Component Δ*θ* calculates the difference in soil moisture between two soil blocks by utilizing a Type 3 pump (flow rate decided by the head difference between the beginning and ending nodes of the pump) [28]. The pump curve of Component Δ*θ* is linear to facilitate such measurement, but the output pumping rate is minuscule so it would not alter the soil moisture in either soil block. Lastly, the output flow from Component D is further throttled by the measured soil moisture difference Δ*θ* by “modulated controls” [28] of SWMM control rules, then multiplied by $\frac{1}{\Delta x}$ (also by SWMM control rules) where Δ*x* is the travel distance from the originating soil block to the next, to produce the unsaturated water flow entering the adjacent soil block. The result equals to $D\frac{\Delta \theta }{\Delta x}$ in Eq 2.

It is worth reiterating that the measurement of Δ*θ* does not alter either soil moisture of the soil block, or the net sum of outflow to the receiving soil block, because the pumping rate of Component Δ*θ* is minimized (the suggested scaling ratio is 10^−9^). All measurements of Δ*θ* throughout the proposed methodology have the same precaution applied.

For vertical downward water movement in Fig 2(B), the only difference is the addition of unsaturated hydraulic conductivity *K*, which does not multiply with the soil moisture gradient $\frac{\Delta \theta }{\Delta z}$ in the calculation of the flow flux as shown by Eq 3. The water flow controlled by *K* is influenced only by gravity (thus it exists only in the vertical direction), which is free draining with a zero gradient (in either soil moisture gradient or matric head gradient) by definition [33]. Therefore, the value of *K* is solely decided by soil moisture of the soil block where the flow originates from, irrelevant to soil moisture of the receiving soil block, because all properties including soil moisture was considered homogeneous within a soil block; therefore, water entering a soil block can be considered immediately distributed evenly in the block. In other words, it would not be necessary to consider how flow moves after reaching a new soil block. This approach was also recommended by models such as GIFMod [23]. Similar to *D* in Fig 2(A), Component K is also simulated by a Type 4 pump with a pump curve delineating the relationship between soil moisture and unsaturated hydraulic conductivity *K* based on Eq 4.

**Fig 3:**
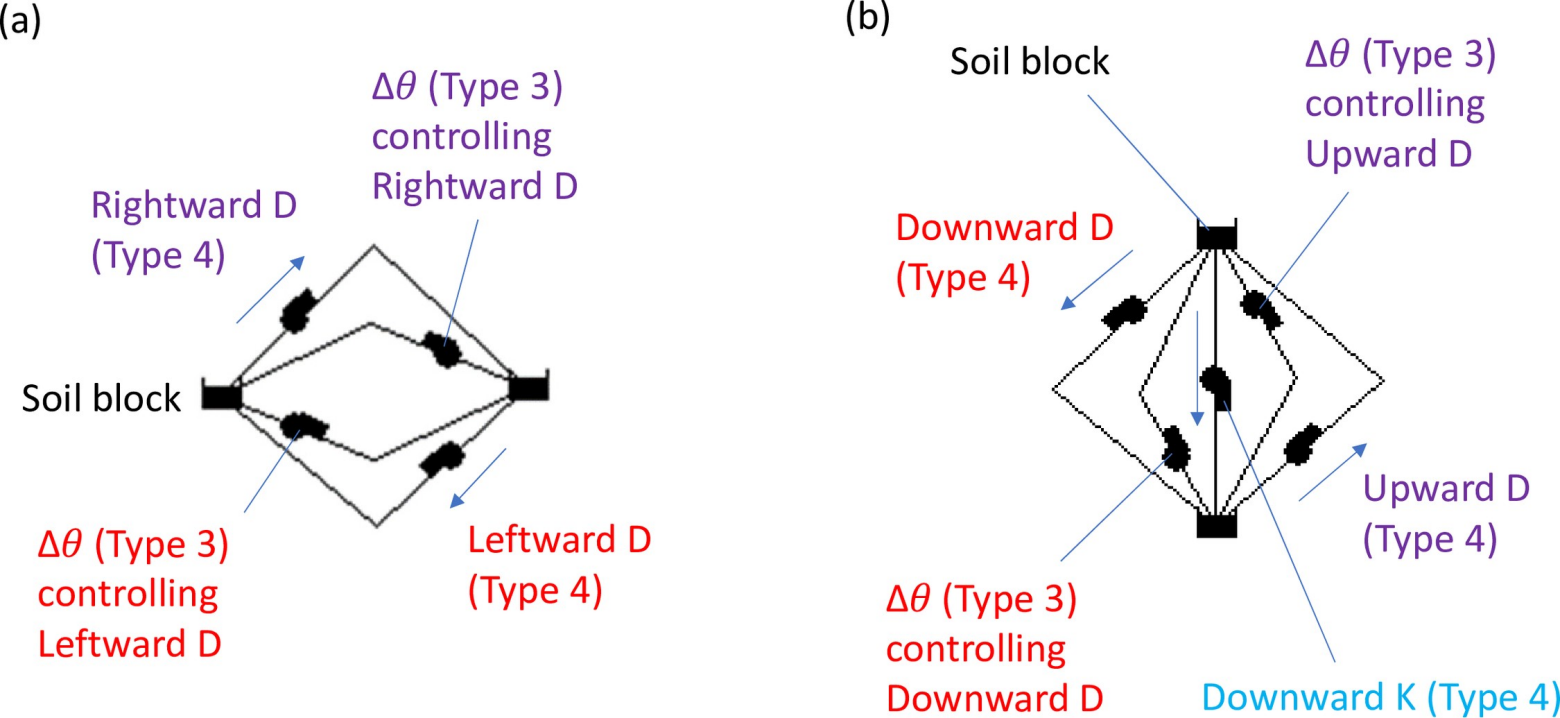
SWMM models simulating horizontal and vertical unsaturated flows.

Models of water movement should allow flow in both directions (i.e., leaving or entering a soil block) even though only one direction would be activated at a time. Allowing flow in both directions enabled the model to simulate dynamic soil moisture distributions as storms progressed. Models are recommended to be constructed two-directionally, as in the examples shown in Fig 3, unless it is known that the flow is only one-directional. Fig 3(A) and 3(B) are the two-directional cases corresponding to Fig 2(A) and 2(B), respectively.

### 2.3. Procedure to simulate the Richards equations for soil blocks with different textures

Even though water movement can be explained by soil moisture gradients, it is ultimately driven by matric head. The water retention curve between soil matric head and water content is different for each soil texture. Therefore, when water travels through different soil textures, the soil moisture difference between different soil textures cannot represent the difference of matric head. In this way, Eqs 2 and 3 do not work across different soil textures.

**Fig 4:**
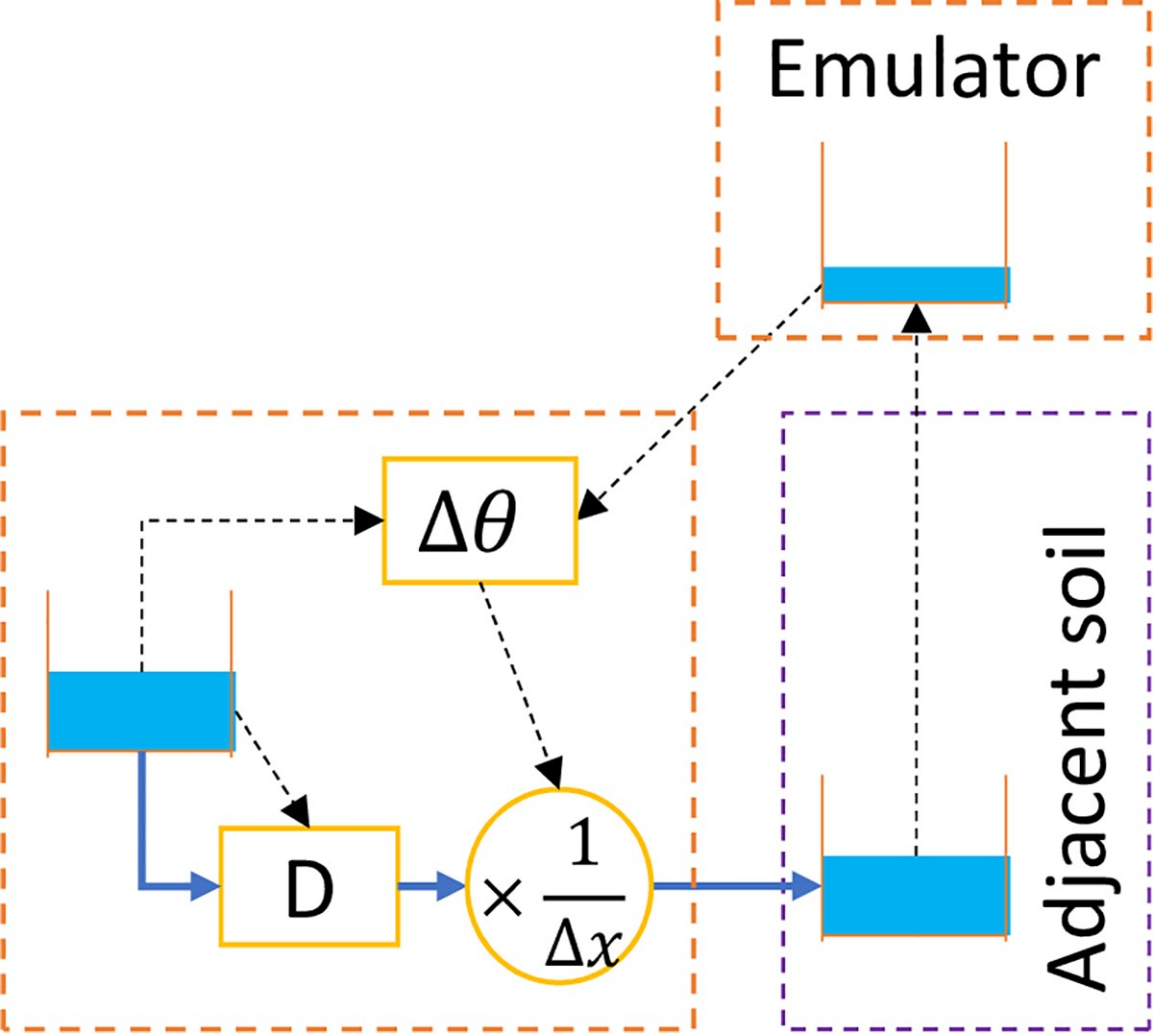
Simulating one-directional horizontal water movement across soil textures with an emulator.

In response to the challenge, the current methodology suggested to use an imaginary soil block called an “emulator” representing the receiving soil block. The matric head of the emulator equaled that of the receiving soil block, but the soil moisture of the emulator was recalculated using the retention curve of the originating soil block. An example of one-directional horizontal water movement across soil texture borders is given in Fig 4. The soil block in the left (orange, thick dashed boundary) and the soil block in the right (purple, thin dashed boundary) are of different soil textures. The emulator converts soil moisture of the purple soil to that of the orange soil with the same matric head. Component Δ*θ* interfaces with the simulated soil moisture in the emulator (top orange soil block with thick dashed boundary) so the flow associated with Eq 2 can be correctly calculated. The flow associated with Component K (Fig 2(B)) does not need an emulator, even if it flows across soil texture boundaries, because it is unrelated to the soil moisture gradient. An emulator takes care of only flow in a single direction, so two emulators are needed to simulate situations in Fig 3(A) or 3(B).

**Fig 5:**
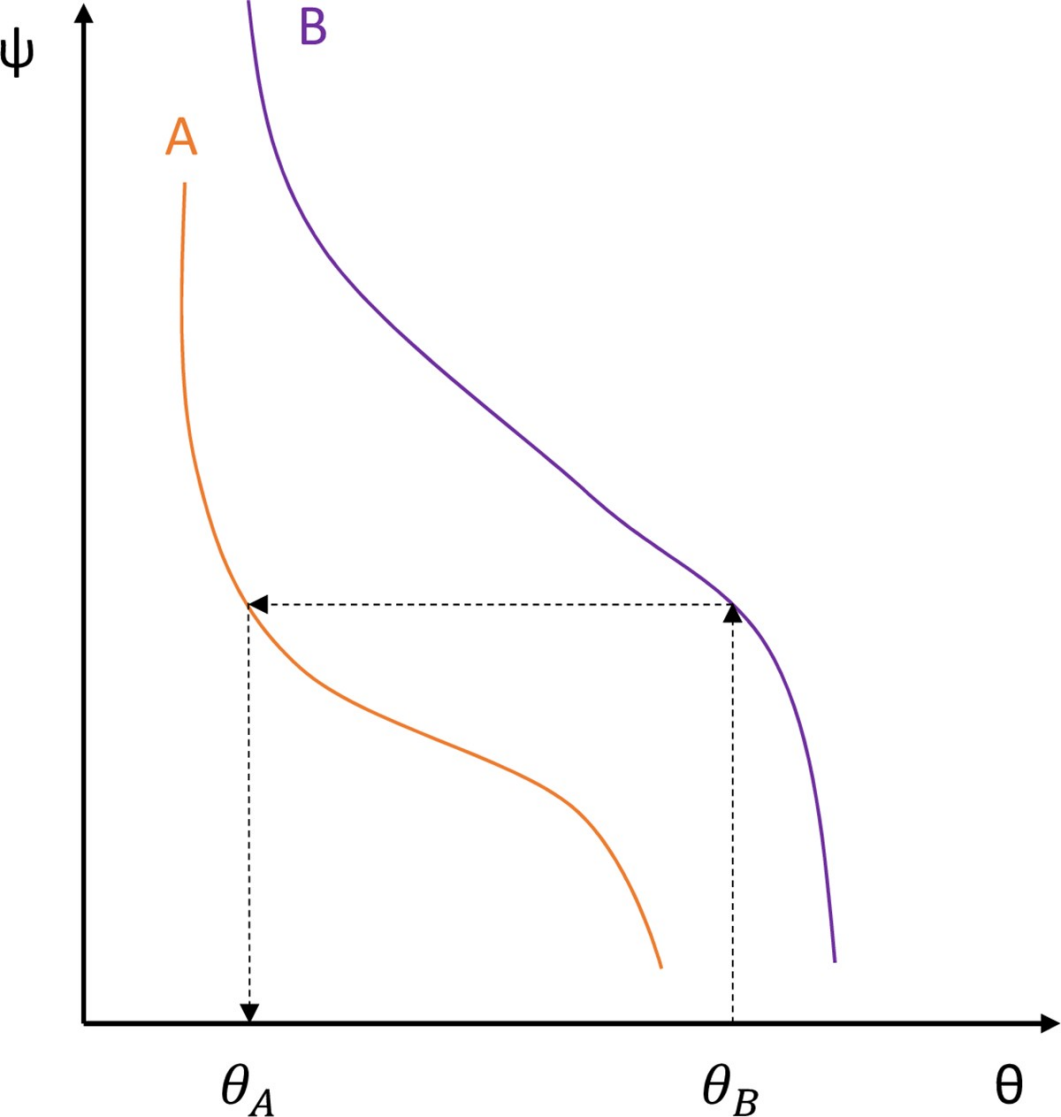
Work process of the emulator.

The theory of an emulator is explained by Fig 5, which shows water retention curves of two hypothetical soils. The horizontal axis is soil moisture, and the vertical axis is matric head (top is negative and zero at the bottom). Soil A is represented by the orange soil (thick dashed boundary) in Fig 4, and soil B is represented by the purple soil (thin dashed boundary). Following the process marked by the dashed arrows, the emulator finds the matric head of soil B first based on the given soil moisture (*θ*_*B*_) and then finds the equivalent soil moisture for soil A (*θ*_*A*_) with the same matric head, which is the soil moisture exhibited by the emulator. Since the relation between *θ*_*A*_ and *θ*_*B*_ is always one to one, an emulator essentially operated on a single curve between *θ*_*A*_ and *θ*_*B*_ if the water retention curves of soils A and B were known.

**Fig 6:**
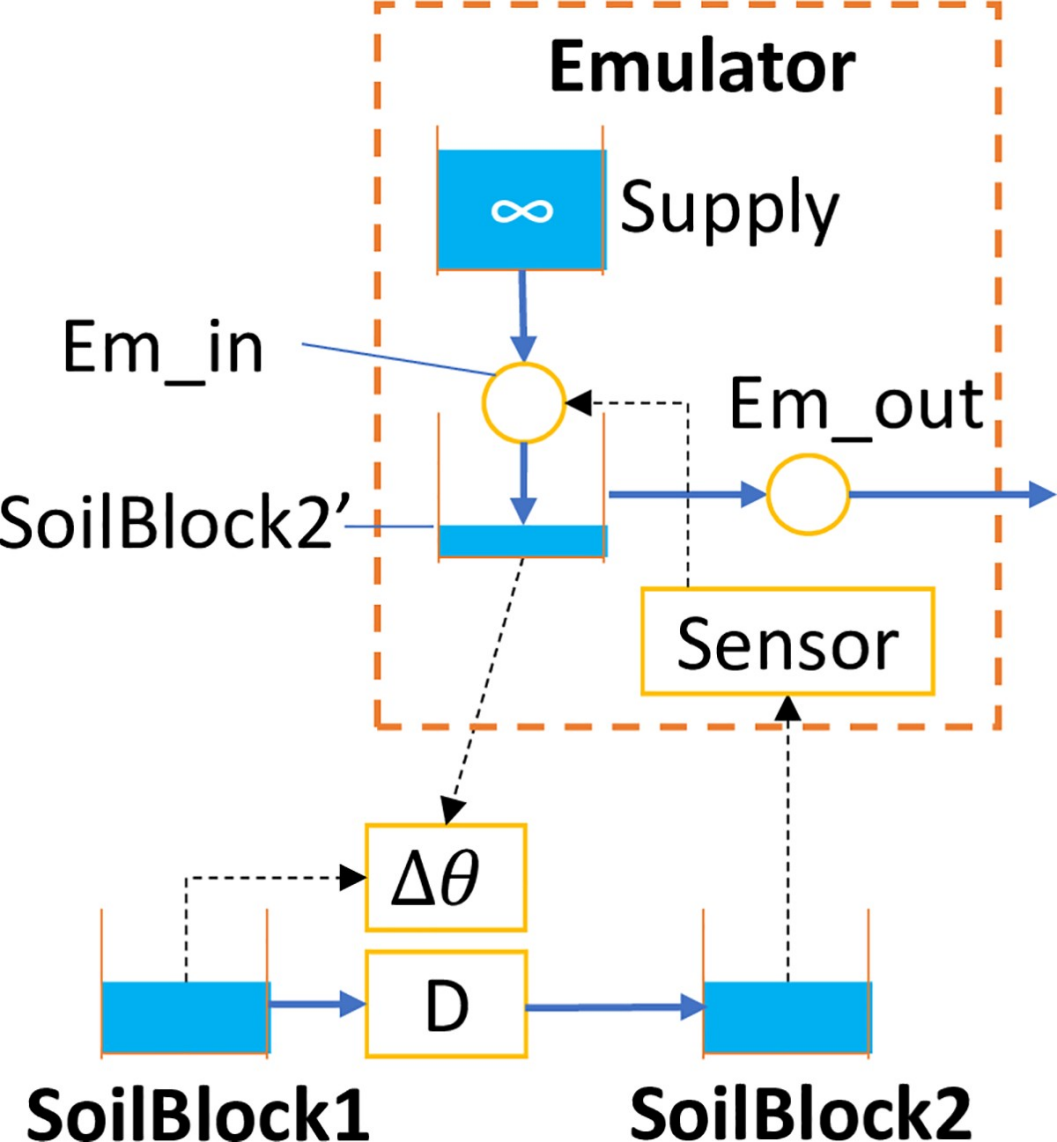
Mechanism of an emulator in one-direction horizontal D flow.

The details of the emulator are shown in Fig 6 with the originating soil block “SoilBlock1” and the emulator (enclosed by a dashed orange box). SoilBlock1 and SoilBlock2 have different soil textures. The emulator converts the soil moisture of SoilBlock2 to simulated soil moisture in SoilBlock2’. SoilBlock2 and Soilblock2’ have the same matric head but different soil moisture, as Soilblock2’ and SoilBlock1 have the same water retention curve. There should be more than one way to design the emulator to achieve the same purpose, but Fig 6 illustrates one way that was proven to work.

The key component in the emulator is the sensor, which is simulated by a Type 4 pump. It adopts a pump curve converting soil moisture of SoilBlock2 to that of SoilBlock1 with no change in the matric head by the principle discussed in Fig 5. Such a pump curve can be created by the assistance of an EXCEL tool created for the implementation of the methodology (see S1 Data). The actual pumping rate of the sensor must be reduced (by control rules) to be minuscule so the soil moisture of SoilBlock2 is not altered by such measurement. The reduced pumping rate is restored at the pump Em_in in the emulator by modulated controls of SWMM control rules [28]. Em_in draws water from a very large water supply which is independent of the rest of the model. The pumping rate of Em_in is converted to water depth in SoilBlock2’ by the outlet link Em_out, which adopts a rating curve with a 1:1 linear relation between values of the water depth and the flow rate and accomplishes the soil moisture conversion from SoilBlock2 to SoilBlock2’ in Fig 6. While the emulator had a complicated structure, most parameters of components of the emulator had fixed values so templates can be used, which were provided in the S2 Data.

## 3. Results

### 3.1. Performance comparison with HYDRUS

**Fig 7:**
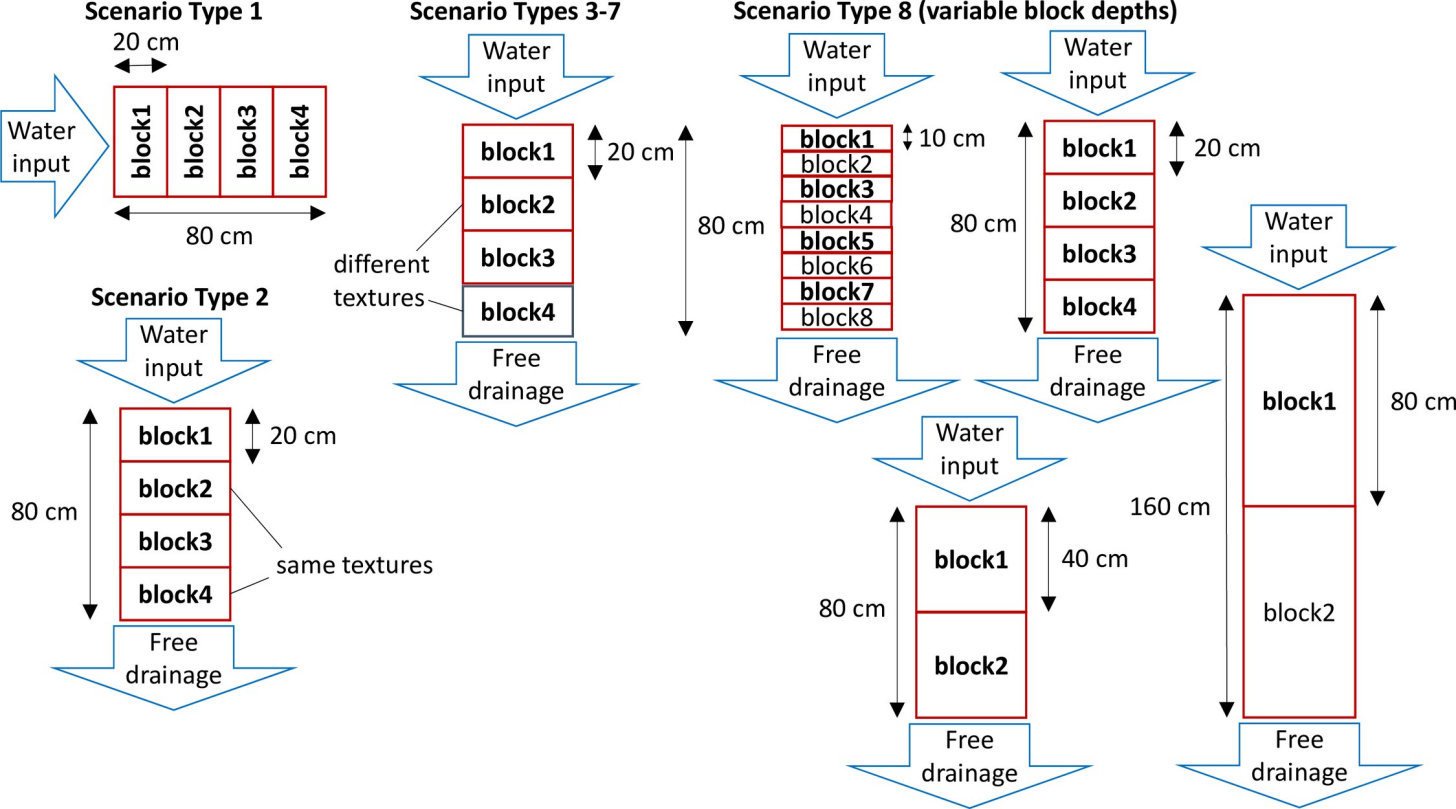
Physical setups of the test scenarios (layers in bold are examined).

The proposed methodology was compared to HYDRUS 1-D for eight GI storm response scenarios shown in Fig 7 and Table 1. HYDRUS is a popular unsaturated zone hydrology model that incorporates the complete Richards equation and Van Genuchten relation and solves the equations numerically by the finite element method [33]. Therefore, HYDRUS was utilized by this research as the benchmark reference. These eight scenarios were used to test the methodology under different simulation challenges to show its robustness. The soil moisture results of HYDRUS-1D were averaged from five observation points to represent the mean soil moisture of a soil block, thus results were comparable to the proposed SWMM methodology.

**Table 1:** Summary of test scenarios.

Type	Soils	Length of each block (cm)	Initial condition	Δx or Δz	Mean error in soil moisture				Max error in soil moisture			
1	Uniform sand	20	Moisture: 0.3	Half length	< 5e-3				< 5e-3			
	Uniform loam				< 5e-3				0.01	<5e-3	<5e-3	0.01
	Uniform Silt				< 5e-3				0.01	0.01	0.01	<5e-3
	Uniform clay				< 5e-3				0.01	0.01	0.01	<5e-3
	Uniform sand			Full length	< 5e-3				< 5e-3			
	Uniform loam				< 5e-3				< 5e-3			
	Uniform Silt				< 5e-3				< 5e-3			
	Uniform clay				< 5e-3				0.01	0.01	0.01	<5e-3
2	Uniform sand	20	Moisture: 0.3	Half length	0.01	0.01	<5e-3	<5e-3	0.03	0.02	0.01	<5e-3
	Uniform loam				< 5e-3				0.01	0.01	<5e-3	0.01
	Uniform Silt				0.01	<5e-3	0.01	0.01	0.02	0.02	0.02	0.03
	Uniform clay				< 5e-3				0.01	0.01	0.02	0.02
	Uniform sand			Full length	0.01	0.01	<5e-3	<5e-3	0.02	0.01	0.01	<5e-3
	Uniform loam				< 5e-3				< 5e-3			
	Uniform Silt				< 5e-3				0.01	0.01	0.01	0.01
	Uniform clay				< 5e-3				0.01	<5e-3	0.01	0.01
3	Blocks 1–3: loam Block 4: sand	20	Moisture: 0.3	Full length	<5e-3	<5e-3	0.01	0.01	<5e-3	0.01	0.02	0.02
4	Blocks 1–3: sand Block 4: loam	20	Moisture: 0.3	Full length	0.01	<5e-3	<5e-3	<5e-3	0.02	0.02	0.06	0.01
5	Blocks 1–3: clay Block 4: sand	20	Moisture: 0.3	Full length	<5e-3	0.01	0.01	0.01	<5e-3	0.02	0.01	0.02
6	Blocks 1–3: sand Block 4: clay	20	Moisture: 0.3	Full length	0.01	0.02	0.05	<5e-3	0.04	0.05	***0*.*10***	0.02
7	Blocks 1–3: sand Block 4: loam	20	Head: -0.15 m	Full length	<5e-3	<5e-3	0.01	<5e-3	<5e-3	<5e-3	0.01	<5e-3
	Blocks 1–3: sand Block 4: clay				<5e-3	<5e-3	0.01	<5e-3	0.01	0.01	0.04	<5e-3
8	Uniform loam	10	Moisture: 0.3	Full length	<5e-3				0.002^1^			
		20			<5e-3				0.003^1^			
		40			<5e-3				0.005^1^			
		80			<5e-3				0.007^1^			

Errors between 0.05 and 0.1 and larger than 0.1 in soil moisture are underlined and in italicized bold, respectively.

^1^ Averaged from examined layers (Fig 7).

The mean was calculated to show the cumulative model bias:

$$\mu =|\frac{\sum_{1}^{n}\left({\theta_{n,m}-\theta }_{n,o}\right)}{n}|$$ (6)

where *μ* is the mean value, *n* is the number of value pairs (observed vs. modeled), *θ*_*n*,*m*_ is the *n*-th modeled value, and *θ*_*n*,*o*_ is the *n*-th observed value.

The mean and maximum absolute errors are given (Table 1) instead of the coefficient of determination (R^2^) or Nash-Sutcliff Efficiency (NSE) because most results were very close and fluctuate in a narrow range of approximately 0.1–0.4. For scenarios with larger errors, absolute errors (to decide the trustworthiness of the methodology at the particular conditions) and conditions when such larger errors happen provide more useful information than R^2^ or NSE.

In Fig 7, only soil blocks in bold were evaluated. All contact (i.e., flow cross-sectional) area between soil blocks is 10 square meters in SWMM, and all water input has a constant flux of 10^−7^ m/s (approximately 3.6 mm/hr), which was reduced in Scenario 1 due to HYDRUS numerical convergence issues. Parameters of soil water retention curves from the HYDRUS default library were used. In all scenarios, because the free drainage boundary condition is defined in HYDRUS as a zero-gradient boundary condition [33], the methodology simulated the boundary condition by Eq 3 with a zero soil moisture gradient (i.e., only hydraulic conductivity K exits).

In Scenarios 1 and 2, Δx of half and full lengths of the soil block in the flow direction were both tested. Scenarios 3–6 tested the methodology for water crossing soil layers of different textures. Scenario 7 tested the initial conditions of equal matric potential, instead of equal soil moisture. Lastly, Scenario 8 tested the effect of the soil block dimension in the flow direction with a single soil texture of loam. Scenarios 1, 2, and 8 relate to recommended model setup parameters and their discussions were combined in Section 4.1. Discussions for other scenarios were included in Section 4.2.

### 3.2. Field comparison with HYDRUS and SWMM LID module

As an additional test, observation data collected from a rain garden on the campus of Villanova University (18 km west of Philadelphia, Pennsylvania) was utilized to compare the proposed methodology, the baseline HYDRUS model, and the LID module in SWMM. The rain garden has an area of 230 square meters and a 1:10 area ratio to the drainage impervious area. The engineered soil (silty sand) in the rain garden has a depth of 1.2 meters with soil moisture measured by HydraProbe (Stevens Water, Portland OR, U.S.A.) at four depths: 10 cm, 35 cm, 65 cm, and 91 cm. The rain garden relies solely on infiltration and does not have an underdrain pipe. The native soil was classified as sandy silt. Models were created for a storm on January 3, 2015 with a total rainfall depth of 49.3 mm. All models started simulations from December 24, 2014 with actual weather data to ensure the simulation results for the storm on January 3, 2015 were as close to the reality as possible. Only one-dimensional vertical water movement was simulated in the models, and the modeled domain was comprised of the 1.2-meter deep rain garden and 0.4-meter deep native soil beneath the rain garden with free drainage as the bottom boundary condition. In this particular application of the proposed methodology, the depth of each soil block was 20 centimeters.

**Fig 8:**
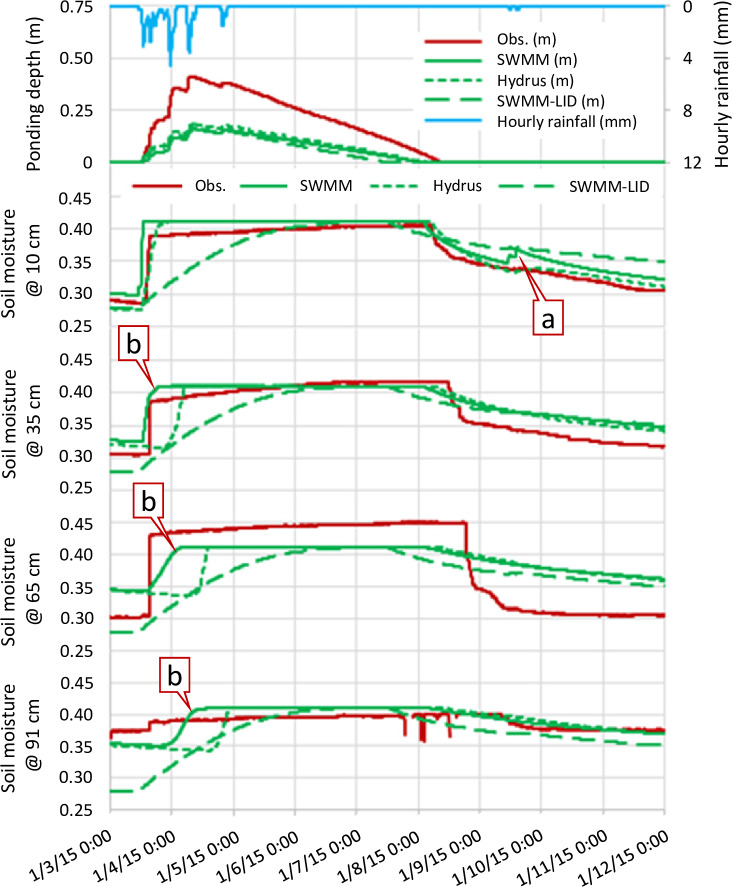
Observed and simulated surface ponding and soil moisture and recorded rainfall data for the storm of January 3, 2015.

The parameters of the retention curves for both soils are provided in Table 2, which were adapted from a master’s thesis at Villanova University [36]. In the SWMM LID module, the LID “bio-retention cell”without a drain system was chosen so the properties of the native soil can be applied as parameters of “storage” in bio-retention cells (Table 3). The observed and simulated ponding depths and soil moisture along with recorded rainfall depth are provided in Fig 8 and summarized in Table 4. Points a and b in Fig 8 will be part of the discussions in Section 4.3. If a large error was caused by a slight (approximately two hours) temporal mismatch of the wetting front, the error excluding the mismatched period was also reported. Soil moisture from the SWMM LID module is identical at all soil depths in Fig 8 because the SWMM LID module considered the whole soil region as a lumped system.

**Table 2:** Soil retention curve parameters.

	*θ*_*r*_	*θ*_*s*_	*α* (1/m)	n	K_s_ (m/s)
Rain garden	0.1	0.41	24.68	1.73	6.1e-7
Native soil	0.1	0.41	0.12	1.12	6.1e-7

**Table 3:** Parameters used by SWMM LID module.

Surface				Soil							Storage			
Bern height (mm)	Vegetation volume	Surface roughness^1^	Slope^1^	Thickness (mm)	Porosity^2^	Field capacity^3^	Wilting point^2^	Conductivity^2^ (mm/hr)	Conductivity slope^4^	Suction head^5^ (mm)	Thickness (mm)	Void ratio^2^	Seepage rate^2^ (mm/hr)	Clogging factor
1000	0	0.1	0	1200	0.41	0.2	0.1	2.20	32.17	24.61	400	0.41	2.20	0

^1^ Dummy, default values used.

^2^ Based on Table 2.

^3^ Little effect on soil moisture and water depth, so the default value is kept.

^4^ Calculated by the SWMM user’s manual [28] based on the soil composition tested by Zukowski et al. [37].

^5^ Calculated from Table 2 [38].

**Table 4:** Summary of error compared to observation data for the methodology, HYDRUS, and SWMM LID module.

	Proposed methodology		HYDRUS		SWMM LID module	
	Mean error	Max. error	Mean error	Max. error	Mean error	Max. error
Ponding depth^1^ (m)	0.079	0.251	0.070	0.222	0.083	0.242
Soil moisture @ 10 cm	0.015	0.123^2^	0.007	0.045^4^	0.005	0.094
Soil moisture @ 35 cm	0.014	0.088^3^	0.007	0.059	0.005	0.088
Soil moisture @ 65 cm	0.004	0.082	0.001	0.098	0.018	***0*.*134***
Soil moisture @ 91 cm	0.004	0.052	<5e-4	0.047	0.020	0.094

Errors between 0.05 and 0.1 are underlined and larger than 0.1 in soil moisture are in bold and italicized.

^1^ Higher errors between the simulated and observed ponding depths because of basin dimensions and vegetation volume

^2^ Max error is 0.031 if ignoring the approximately 2-hour period of mismatched wetting fronts

^3^ Max error is 0.035 if ignoring the approximately 2-hour period of mismatched wetting fronts

^4^ Max error is 0.017 if ignoring the approximately 2-hour period of mismatched wetting fronts

## 4. Discussion

### 4.1. Recommended setup parameters related to soil block dimensions

**Fig 9:**
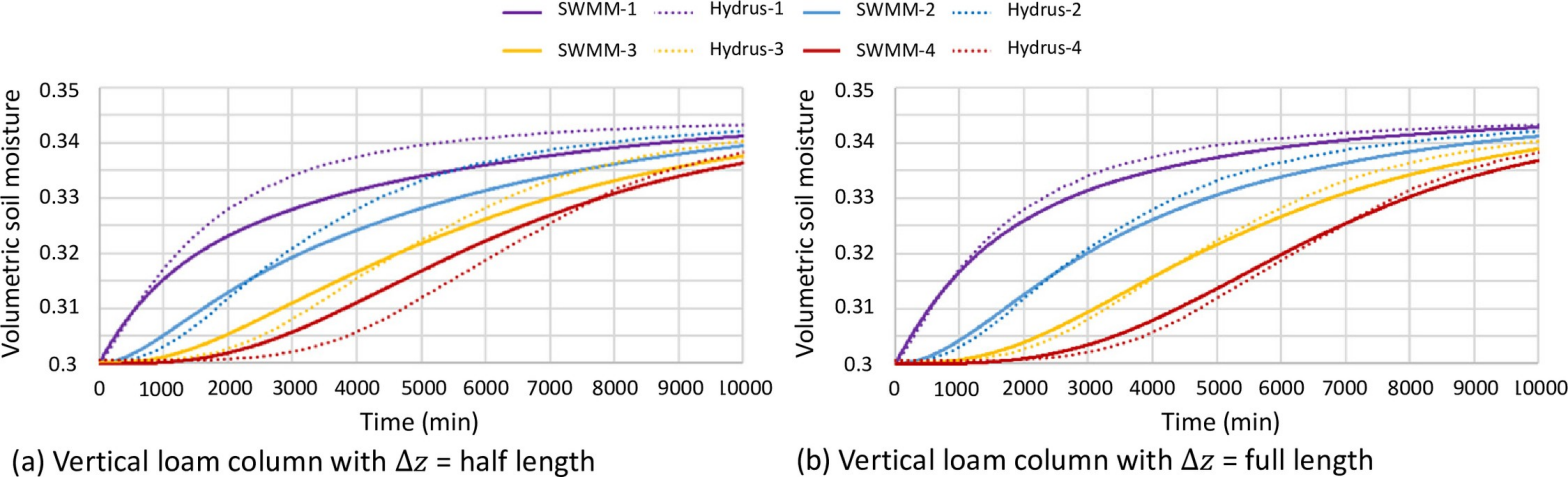
Comparison of vertical loam columns (Scenario 2) with different choices of Δ*z* while “SWMM-1” means the result using the proposed methodology for Soil block 1, and “Hydrus-1” means HYDRUS result for Soil block 1, etc.

Results from Scenarios 1 and 2 (Table 1) included results for different lengths of Δx or Δz in cases of horizontal and vertical uniform soil columns, respectively. In general, the results using the full length of the soil block in the flow direction as Δx or Δz yielded an error up to 50% lower. This difference is illustrated in Fig 9 using an example of the vertical loam in Scenario 2.

Based on Section 2.2, it would not be necessary to consider flow after reaching a new soil block, which implied that the simulated travel distance should be only from the center of the originating soil block to the border of the receiving block (i.e., half the length of the soil block at the flow direction). However, the testing results from Scenarios 1 and 2 found that using such a definition of Δ*x* rendered the model less accurate because it biased the calculated soil moisture gradient. This was also illustrated in Fig 9. When Δx of half the length of the soil block is chosen (Fig 9(A)), soil moisture of the upper soil blocks tends to be underestimated, and that of the lower blocks tends to be overestimated, which indicates an overestimation of water flow rate due to an overestimation of the soil moisture gradient. Using the full length of the soil block in the flow direction as Δx (or Δz if vertical) was thus recommended by the proposed methodology.

Scenario 8 investigated the issue of optimal soil block sizes. Results showed a linear relationship between the magnitude of error and soil block dimension, so ideally the dimension should be as small as possible, but it proportionally increased the effort to create and maintain the model. Scenarios 1–7 (all with a soil block dimension of 20 cm) exhibit a maximum error of around 0.1 (Table 1). Considering soil moisture measurements were usually “noisy” with a maximum observation error of about 0.1 even for the more accurate dielectric-type soil moisture sensors [39], modeling error should be no higher than the margin of such measurement noise. Therefore, it was recommended to use 20 cm (or less) as the soil block dimension at the flow direction in the proposed methodology.

### 4.2. Performance comparison with HYDRUS with more than one soil texture

Scenarios 1, 2, and 8 discussed above all considered only one single soil texture. Errors of soil moisture from those scenarios are all less than 0.01, which were satisfactory. Nevertheless, scenarios considering water flow crossing different soil textures (i.e., Scenarios 3–7) yielded higher errors, particularly in Scenarios 4 and 6.

**Fig 10:**
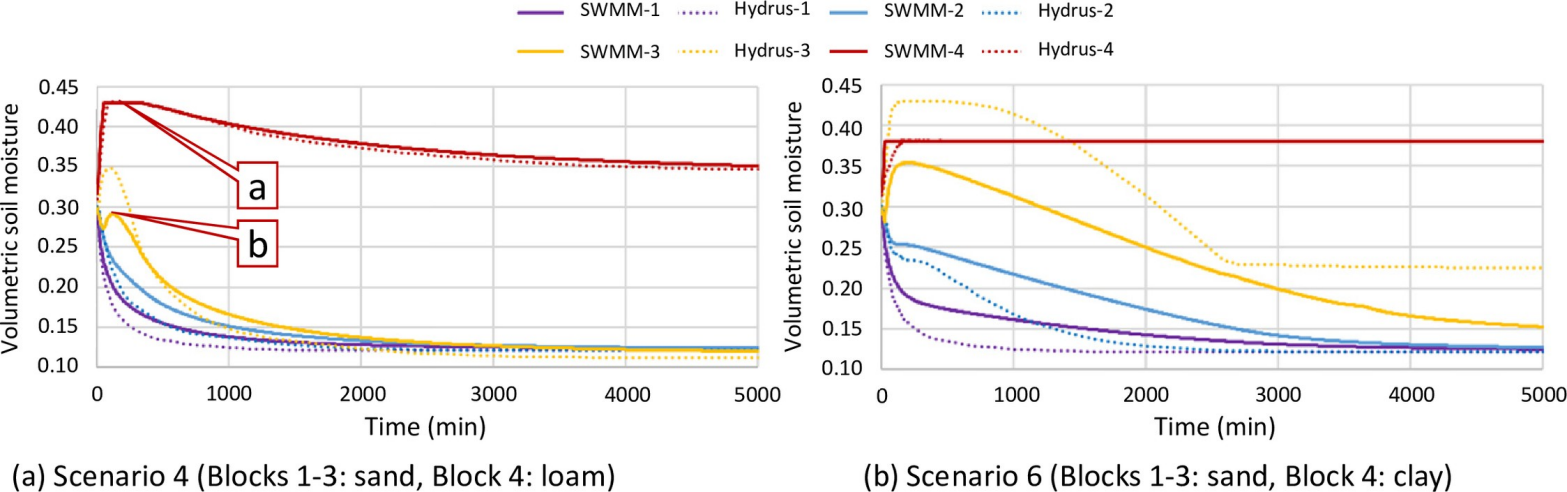
Results from Scenarios 4 and 6.

One thing in common for Scenarios 4 and 6 (Fig 10) was that the bottom soil textures have poorer hydraulic properties than those of the upper layers. In Fig 10(A), the bottom soil block (loam) extracts water from the top soil blocks (sand) very quickly in the beginning of an event simulation due to the initial imbalance of matric head (discussed below), so it reaches saturation quickly as shown by the plateau (Point a). When saturation occurs, Soil block 4 stops receiving water, and the soil block above it (block 3) accumulates water and its soil moisture rebounds (Point b). Both SWMM and HYDRUS results captured such complex dynamics, but Soil block 3 exhibited a larger error in the initial rebound (Point b).

The observations displayed in Fig 10(A) and 10(B) have similar mechanisms to the observations in Fig 9 but those in Fig 10(B) has higher error particularly at Soil block 3. Soil block 4 (Fig 10(B)) is constantly saturated, but that is not necessarily the case for Soil block 3. SWMM could underestimate water flow because the proposed methodology adopted the mean soil moisture gradient between soil blocks. In reality, a sharp soil moisture (as well as soil matric head) gradient can be localized at the border, spurring a higher water flow rate. Fig 10(B) has a higher error likely because the bottom clay layer is saturated for an extended time.

Scenarios 4 and 6 showed that adopting uniform initial soil moisture for all soil layers regardless of the difference in soil texture caused larger errors. Such an initial condition would only be suitable for model testing because it is not a balanced state in the real world. Therefore, Scenario 7 used a uniform initial matric head of -0.15 m for all soil layers in the settings of Scenarios 4 and 6 without other changes. The results showed much lower errors (Table 1). However, Soil block 3 still showed larger (but significantly reduced) error, indicating the detrimental influence of localized high soil moisture/matric potential gradient on the accuracy of the proposed methodology.

### 4.3. Field comparison with HYDRUS and SWMM LID module

There was consistently higher observed ponding depth for the proposed methodology, HYDRUS and the SWMM LID module (Fig 8). This was likely due to how the storage curve was defined in SWMM (i.e. the methodology and the SWMM LID module) and HYDRUS, which assumes a constant surface area vs. depth. However, the basin is constructed like a “bowl” so storage volume is small when the depth is low [40]. Also, the models did not consider the volume of vegetation. Both factors could drive the actual ponding depth higher than the modeled result with constant surface area and no vegetation.

Turning to soil moisture, the result showed that the SWMM LID module was unable to depict the wetting front accurately. Even though it described the general behavior of the system (with low mean bias), it yielded the highest maximum error at all depths among the three models (Table 4) if a slight temporal mismatch of wetting fronts was overlooked. For the proposed methodology and HYDRUS, they generally showed good agreement with each other and with the observation data, particularly at the 10 cm depth, with two exceptions: firstly, results of the proposed methodology were sensitive to small rainfall input (Point a), and secondly, results of the proposed methodology responded to rainfall earlier (Point b) compared to those of HYDRUS. Both can be explained by the assumption of homogeneous soil moisture in soil blocks made by the proposed methodology. Any reported soil moisture from the methodology was the average of the whole soil block, so any rainfall input to the topmost soil block was immediately accounted for, which explained Point a. Point b can be explained similarly because HYDRUS simulated soil moisture at an exact nominal depth, but the methodology detected the mean soil moisture of the whole imaginary soil block containing the nominal depth.

It was intriguing to see that the observed soil moisture data responded earlier than that in HYDRUS. In the observation data, the temporal differences in the initialization of responses among different depths were extremely short, indicating a fast downward water intrusion in the field. Both can be explained by the existence of preferential flow [41], so HYDRUS’ classical single-porosity van Genuchten relation can underestimate the downward water movement speed in real settings, as Fig 8 shows. Despite these discrepancies, the methodology showed good agreement with the observation data, and on par with HYDRUS in accuracy. Therefore, the methodology can be trusted in predicting soil moisture in GI in the field.

## 5. Conclusion

Based on equations to predict soil water diffusivity and unsaturated hydraulic conductivity for soil moisture, a methodology to simulate the unsaturated zone hydrology in SWMM was proposed. Flow governed by soil water diffusivity was calculated according to the mean soil moisture gradient between soil blocks, and flow governed by unsaturated hydraulic conductivity was calculated based on the value of soil moisture of the soil block where the flow originated from.

The methodology was compared to HYDRUS as a baseline model using theoretical soils data, and the discrepancy between the two models was generally small. A large discrepancy only happened when high contrasts in soil moisture (also matric potential) exist at the soil texture border, which was usually caused by a constantly saturated soil layer with low hydraulic properties at the bottom. Such discrepancy was caused by the use of a linear soil moisture gradient to approximate the Richards Equation. Users utilizing the proposed methodology should exercise caution about the results when such a condition exists. However, the error was only significant immediately above the soil texture border and the results can still be considered accurate for other layers, particularly when the length of each soil layer was less or equal than 20 cm.

The methodology was further compared with both HYDRUS and the SWMM LID module using field data. The performance of the proposed methodology and HYDRUS were very similar, indicating the feasibility to use the proposed methodology on field data. The SWMM LID module can capture the trend of soil moisture, but it had lower accuracy compared to the proposed methodology and HYDRUS. The field test revealed that the methodology reported soil moisture changes (i.e., wetting front) slightly earlier than HYDRUS did, probably because the methodology detected soil moisture change for a whole imaginary soil block based on the assumption of homogeneous soil moisture.

Overall, the proposed methodology provided accurate estimates of soil moisture distribution in soil and proved that the mechanism of unsaturated zone hydrology can be simulated with high accuracy by using existing components of SWMM without the need to alter the source code of SWMM. As work progresses, several elements can be addressed as future research directions. Firstly, the performed tests adopted the free drainage boundary condition, which is a common boundary condition for GI applications. Broader applications of the proposed methodology by deriving and validating ways to simulate other boundary conditions would be useful. Secondly, the top-most soil block responded to surface water input immediately, which made the estimation of wetting fronts less accurate as the field test showed. To reduce this error, a user can simply use thinner soil blocks to isolate this effect at the “skin” on top or devise a delaying mechanism for water input, which is recommended for future developments of the proposed model. Lastly, the proposed methodology adopted the single-porosity van Genuchten relation. As the field application showed, the preferential flow might play an important role in the field, so it is recommended to introduce dual-porosity models in the proposed methodology in the near future.

## 6. Application guide and assistant tool

A detailed step-by-step application guide and an assistant EXCEL tool (for automatic generation for pump curves of D, K, and the sensor) have been included in the S1 Data and S1 File accompanying this paper.

## Supporting information

S1 File (PDF)

S1 Data (XLSM)

S2 Data (XLSX)
