# Supplementary material for: Methodology to simulate unsaturated zone hydrology in Storm Water Management Model (SWMM) for green infrastructure design and evaluation
Source: PLoS One. 2020 Jul 6;15(7):e0235528. doi: 10.1371/journal.pone.0235528 (PMC7337332; doi:10.1371/journal.pone.0235528)
Supplement: S1 File — (PDF) [file pone.0235528.s001.pdf]

Application Guide to accompany "Methodology to Simulate Unsaturated Zone Hydrology in Storm Water Management Model (SWMM) for Green Infrastructure Design and Evaluation"

A detailed step-by-step application guide of the proposed methodology is provided in this document. An assistant EXCEL tool which automatically generates the pump curves for D, K, and the sensor (in the modulator) can be found among the supporting information files associated with the above-identified article. The first part of this application guide describes parameterization, and the last part provides a recommended procedure to construct a large model.

Fig 1 shows the detailed SWMM setup of the soil blocks and pumps (D, K, and $\Delta\theta$). All parameters different from SWMM default are enclosed by red or green boxes. Values in red boxes were decided by the user, and values in the green box were fixed. Users should choose an appropriate name for individual components.

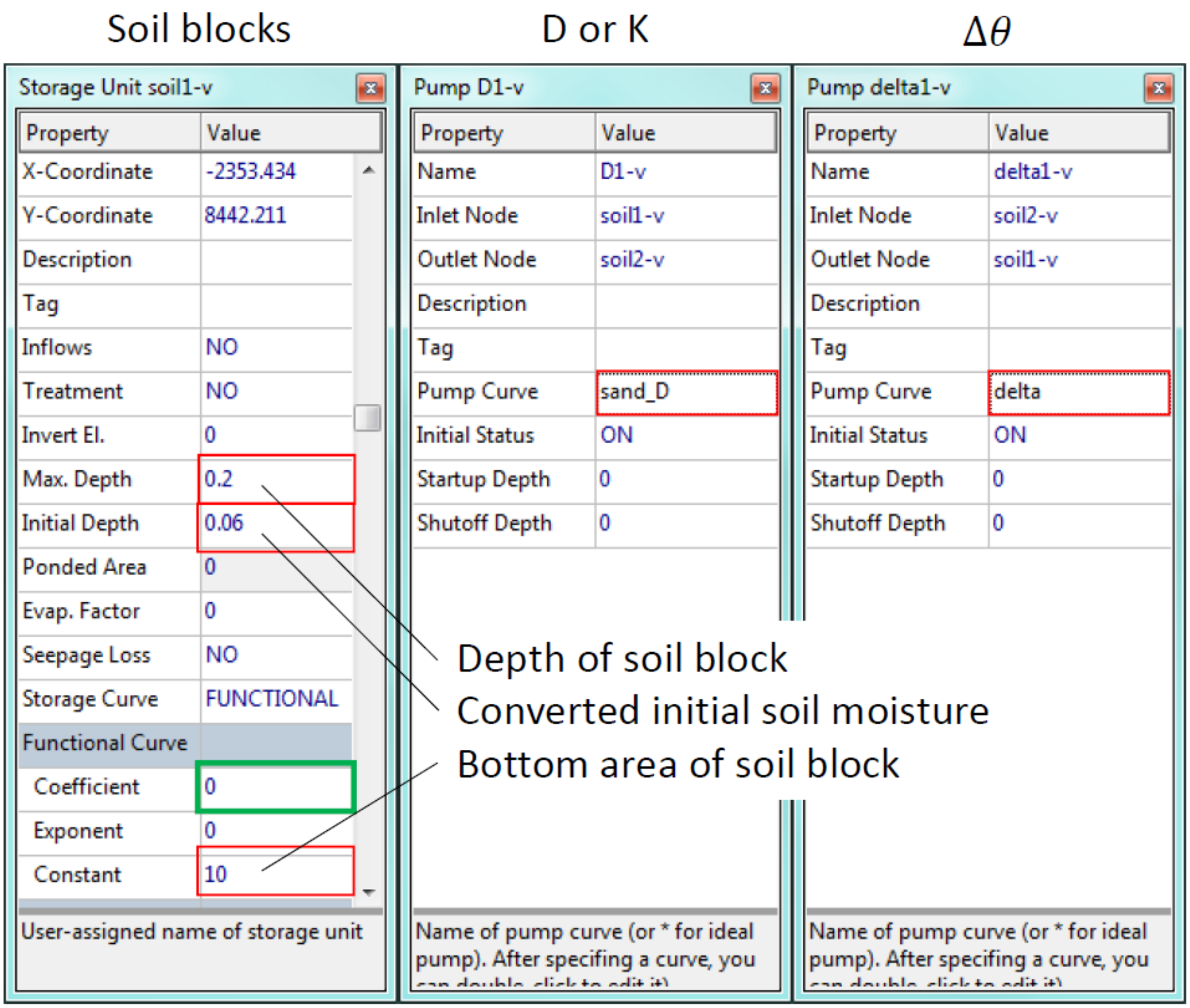

Fig 1. Detailed setup of soil blocks and pumps

For the soil blocks, three parameters need inputs: the maximum depth of the storage, the initial depth of the storage, and the constant of the functional storage curve. The proposed methodology considered a soil block as a storage, and soil moisture was represented by the equivalent water depth in the storage. Therefore, the parameter "Max. Depth" equals the depth of the soil block, and the parameter "Constant" equals to the bottom area of the soil block. The parameter "Initial Depth" represents the actual water depth in the soil block based on the initial soil moisture. In other words, if the soil block is 0.2 meters tall, and the initial soil moisture is 0.3, "Initial Depth" should be 0.06 (=0.2 x 0.3) meters. This is how all soil moisture values were represented in SWMM based on the proposed methodology.

Appropriate pump curves must be selected for pumps in Fig 1. Exemplary D, K, and $\Delta\theta$ pumps curves are provided in Fig 2 below. The table of Depth vs. Flow defines the relationship between soil moisture and D or K. The $\Delta\theta$ pump scales down the head difference between two soil blocks to miniscule flow (so it will not alter soil moisture) which will be converted back to the actual head difference and used by the Control Rules. An assistant tool (downloadable either from the journal website associated with the paper) was created to assist the creation of D and K pump curves. It works with the flow unit system of CMS in SWMM.

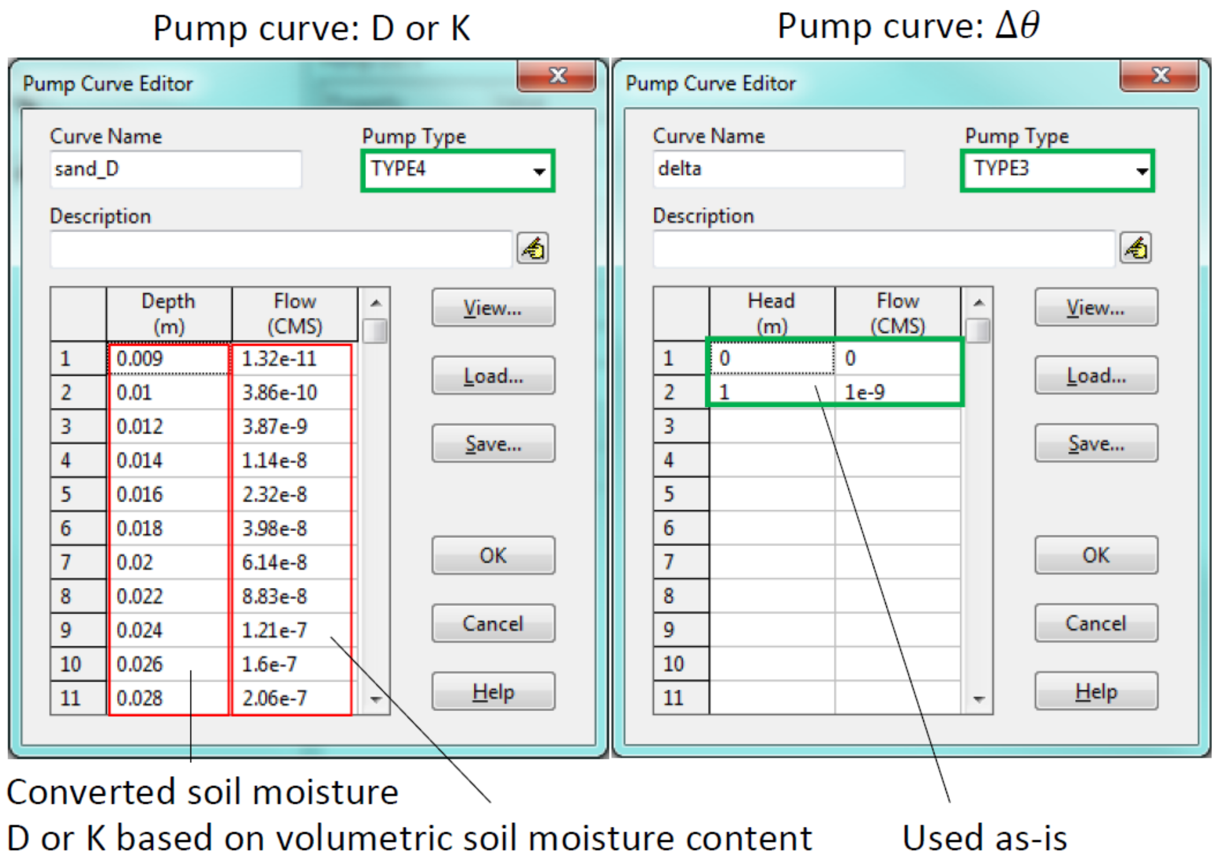

Fig 2. Example pump curves for D (or K) and Delta

There were Control Rules to accompany pump D, pump K, and soil blocks. Each pump K has a simple set of Control Rules (Fig 3). The first rule (Rule K) multiplies the K flow flux (in unit [L/T]) by the bottom area of the soil block (A_{bottom}) to get the flow rate (in unit [L^3/T]). The second rule (Rule K-stop) stops

infiltration when the soil moisture in the soil block is less than or equal to the residual soil moisture θ_r. All soil moisture is labeled with the subscript "converted" to show they are the actual water depth in the soil block storage unit.

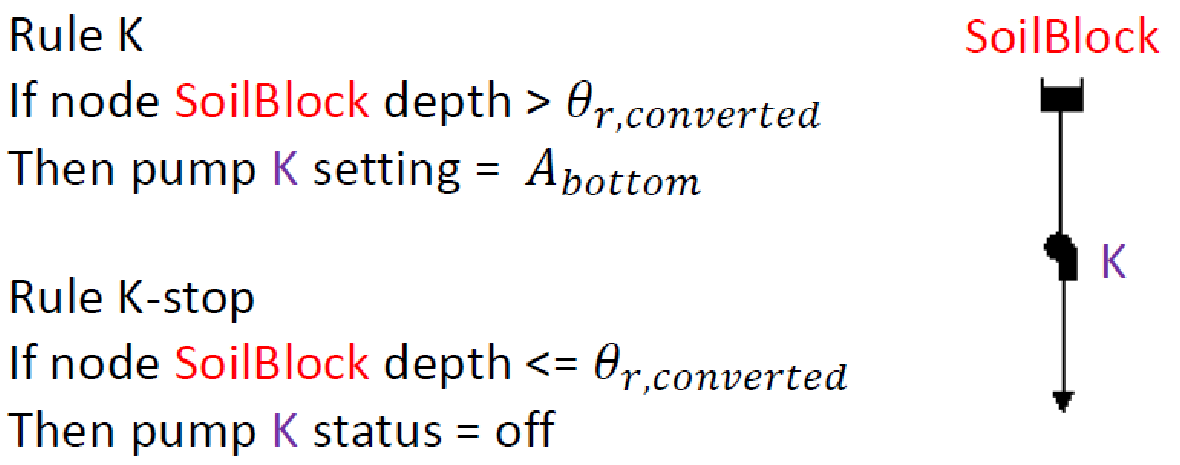

Fig 3. Control Rule (and associated SWMM diagram) for pump K

In most cases, pump D always appeared in pairs to simulate the two-way water flow in real scenarios, so their Control Rules are discussed together, as Fig 4 shows. In Fig 4, pumps delta1 and delta2 represent $\Delta\theta$ pumps associated with D1 and D2, respectively. Horizontal water movement is shown but the identical setup applies to the vertical movement case. Rules D1 and D2 are mirrored so only Rule D1 is explained below. Rule D1 turns on pump D1 when pump delta1 detects higher soil moisture in the left soil block and turns off the pump in the opposite direction. The control curve "Convert" defines how the fractional setting at pump D1 varies with the flow rate of pump delta1 with modulated control. It converts the minuscule flow from a $\Delta\theta$ pump back to its original head difference and further calculates the value of $\frac{\Delta\theta}{\Delta x} \cdot A_{contact}$ from the head difference, so the D flow can be calculated. Therefore, the value C in the control curve (Fig 4) is calculated by Equation 11:

$$C = \left(\frac{1}{Depth\ of\ soil\ block}\right) \cdot \left(\frac{1}{\Delta x}\right) \cdot A_{contact} \qquad (11)$$

In Equation 11, all variables follow the definitions given before, with $A_{contact}$ defined as the contact area perpendicular to the D flow. Δx can be Δy or Δz depending on the flow direction. The first term in Equation 11 converts the head difference to the difference in soil moisture ($\Delta\theta$). As for the second term, it is suggested to use the full length of a soil block in the flow direction produces better results as the published paper delineated.

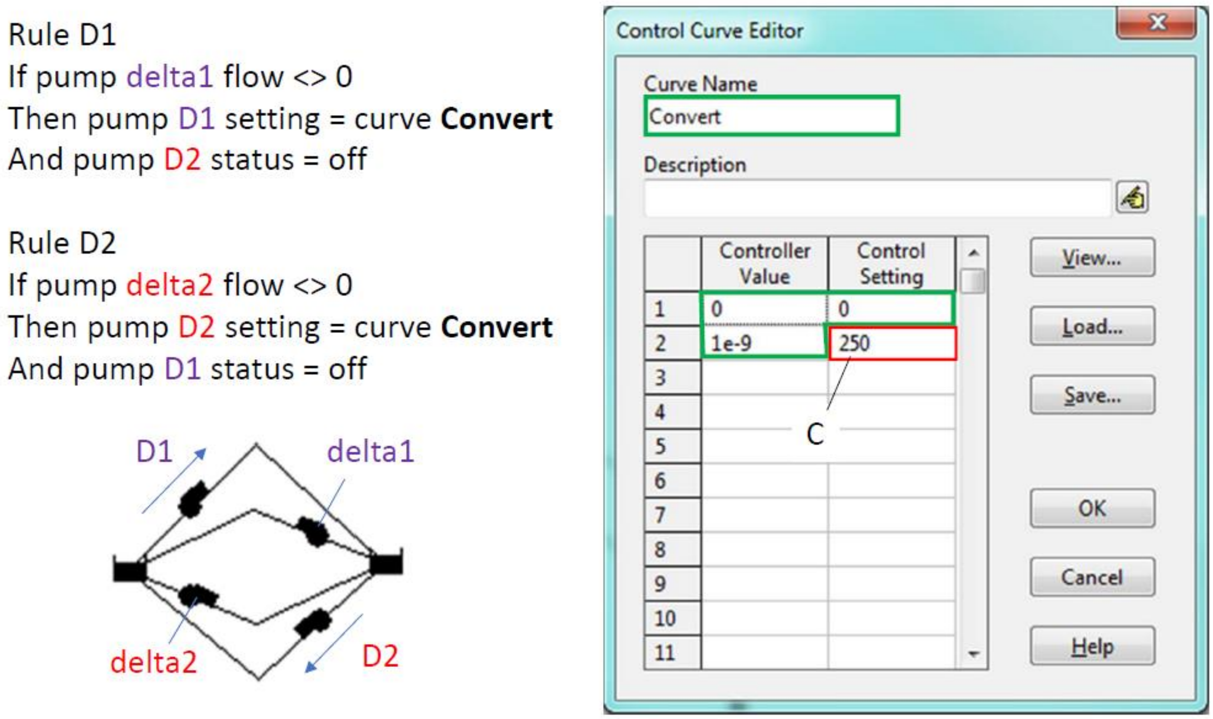

Fig 4. Control Rules (and an associated SWMM diagram and control curve) for pump D

Each soil block has a Control Rule to address soil saturation, as Fig 5 shows. In Fig 5, pumps delta1, delta2, delta 3, and delta 4 represent $\Delta\theta$ pumps associated with D1, D2, D3, and D4, respectively. Fig 5 illustrates a three-block model with D and K flows, and a set of Control Rule associated with the center soil block ("SoilBlock"). A soil block has three default incoming water flows: the K flow (only downward) and two D flows. Rule "Saturation" shuts down all three incoming flows when the soil block is saturated. Rule Saturation has higher priority over other Control Rules that turns on the pumps, so it is given a priority number.

Unlike other Control Rules, Rule Saturation has some flexibility because the three incoming flows do not always exist simultaneously. For example, the very top soil block of a soil column does not have a K flow flowing into it. Users should modify Rule Saturation for each soil block according to its actual conditions.

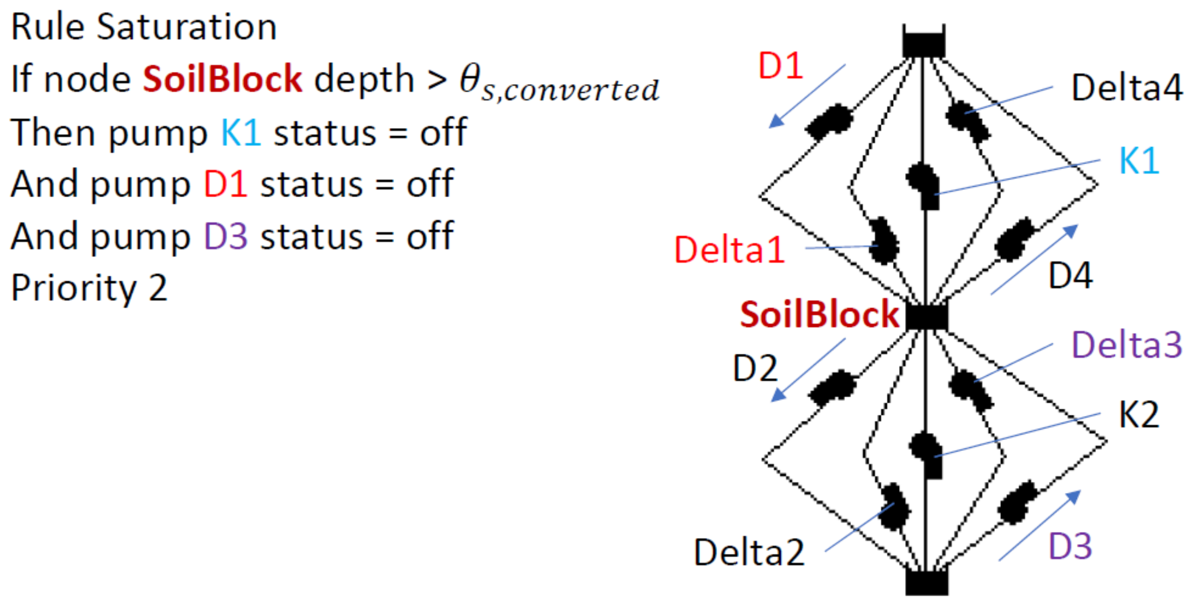

Fig 5. Control Rules for shutting off incoming flows (and an associated SWMM diagram)

<u>Flow across different soil textures</u>

The case with more than one soil textures will be discussed below. Fig 6 in the published article shows the components of a single emulator (enclosed by an orange box), which is associated with the D flow from “SoilBlock1” to “SoilBlock2”. SoilBlock1 and SoilBlock2 have different soil textures. SoilBlock1 and SoilBlock2’ have the same soil texture. Mechanisms outside the emulator block are simplified. The components “SoilBlock2”, “EM_in”, and “Sensor” are color-coded to be used in figures later.

The emulator converts the soil moisture in SoilBlock2 to simulated soil moisture represented by SoilBlock2’. SoilBlock2 and Soilblock2’ have the same matric head but different soil retention curves. The delta block works with SoilBlock2’. The numerical value (not considering units) of the flow rate of pump Em_in is controlled by Control Rules to equal to that of the simulated soil moisture in SoilBlock2’.

Pump Em_in draws water from a very large reservoir (“Supply”) that can be considered infinite. The valve Em_out is represented by an outlet link in SWMM. Em_out simply imposes a 1:1 relationship between the numerical values of the flow rate of pump Em_out and the water depth (i.e. soil moisture in Soilblock2’). Since the value of flow rate is controlled to equal the value of soil moisture of SoilBlock2’, utilization of Em_out sets the simulated soil moisture in SoilBlock2’.

A typical SWMM setup is presented in Fig 6 with D flows between two soil blocks of different soil textures. Emulators are enclosed by orange boxes. The following discussion will be based on the emulator in the upper right corner as the other emulator has an identical structure.

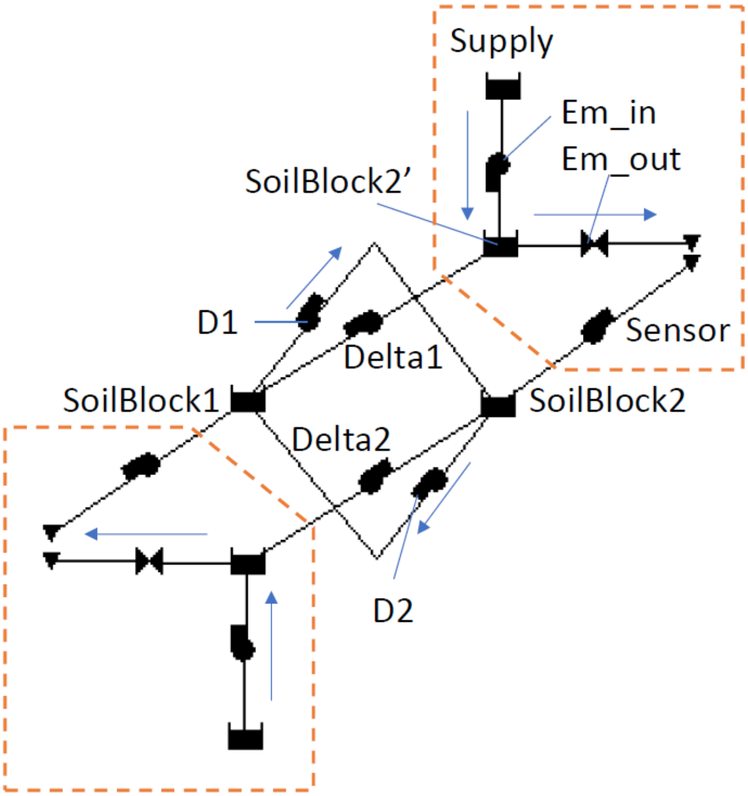

Fig 6. SWMM setup of two-way D flows with associated emulators

Templates for most components of emulators (except for Sensor and associated Control Rules) are provided in Fig 7 and Fig 8. The storage unit Supply mimics an infinite water supply, so it has a huge

surface area and depth. The constant flow rate for pump Em_in is to be regulated by the Control Rules. The bottom area of storage SoilBlock2’ is set at 5 square meters to avoid either slow or abrupt changes in water elevation. Finally, the rating curve of outlet link Em_out imposes the 1:1 relationship between the numerical values of the flow rate and the water depth.

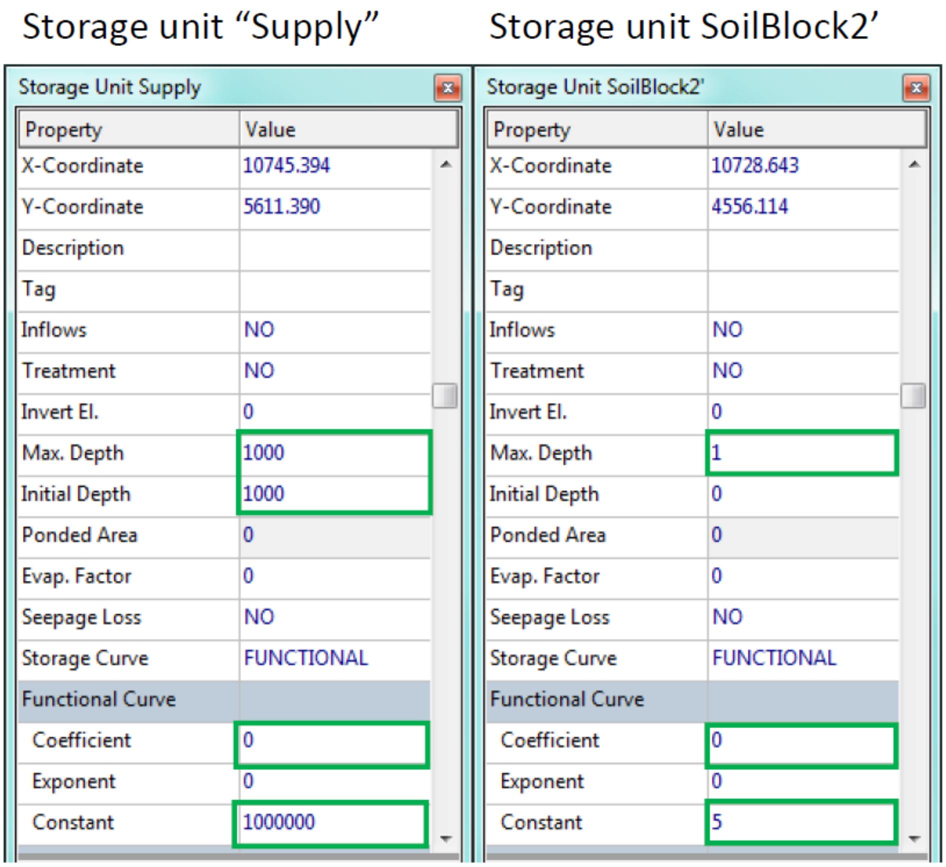

Fig 7. Templates for the two storage units of an emulator

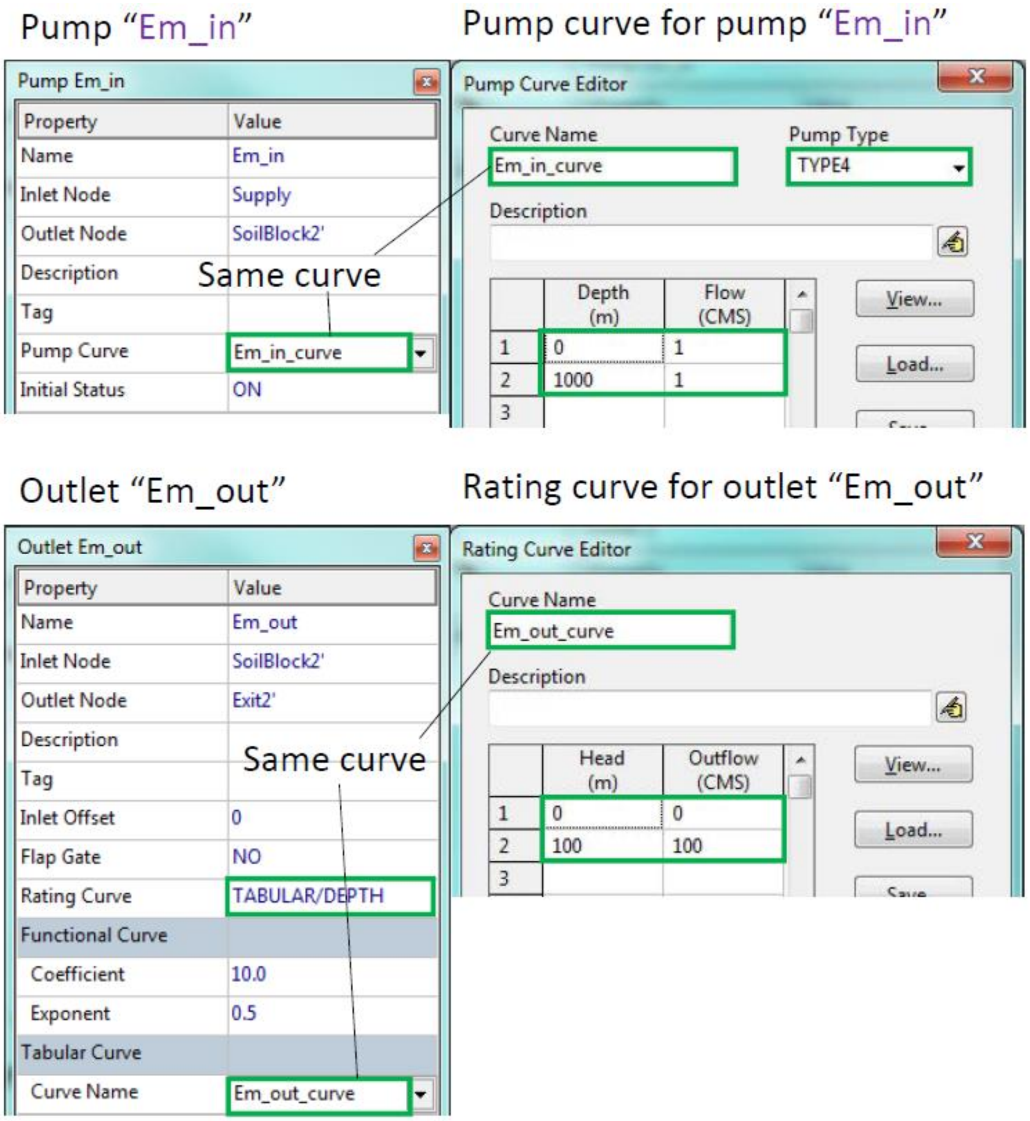

Fig 8. Templates for the pump and outlet units of an emulator

The setting of Sensor and associated Control Rules are discussed in Fig 9. The pump curve for Sensor converts the soil moisture in SoilBlock2 to the equivalent soil moisture in SoilBlock2'. In the pump curve editor, numbers enclosed by red boxes can also be computed by the assistant tool mentioned before.

Similar to the Delta pumps, the flow rate of pump Sensor must draw only a minuscule amount of water from SoilBlock2. This is done by the Control Rule "Downsize". The next Control Rule ("Convert_shutoff")

shuts off the Sensor when the soil is dry. The last Control Rule ("Restore") restores the soil moisture sensed by the Sensor and makes the flow rate of Em_in to match its value.

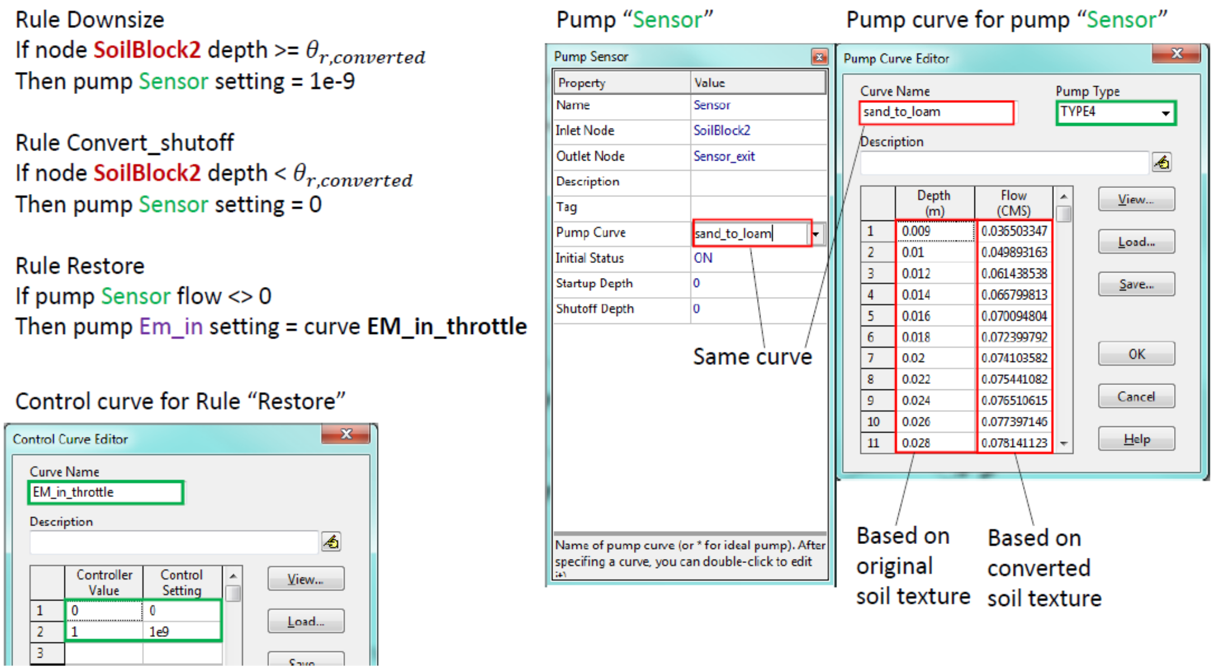

Fig 9. Setup and related Control Rules for "Sensor"

Suggested Application Procedure

The following step-by-step procedure to build a new model from scratch was recommended. It was encouraged to use the assistant tool (downloadable from the journal website associated with the paper) to create D, K, and the Sensor pump curves.

1. Creating pump curves of D, K, delta (Fig 2) and control curve "convert" (Fig 4, ignoring the associated control rules for now) in SWMM;

2. Creating soil blocks and parameterizing them (Fig 1);

3. Linking soil blocks with D, K, and delta pumps as appropriate and parameterizing them (Fig 1);

4. Creating control rules associated with D (Fig 4), K (Fig 3), and soil blocks (Fig 5);

Steps 5-10 would only be required when water was expected to flow through more than one soil textures. For simple models containing only one soil texture, the user was directed to step 11.

5. Creating pump curves for Em_in (Fig 8) and “sensor” (Fig 9), rating curve for Em_out (Fig 8), control curve “Em_in_throttle” (Fig 9), and ignoring the control rules for now;

6. Using Fig 6 as the reference, creating storages for Supply and converted soil moisture (i.e. SoilBlock2’ in Fig 6) and parameterizing them (Fig 7);

7. Using Fig 6 as the reference, creating pump Em_in and outlet Em_out and parameterizing them (Fig 8);

8. Using Fig 11 as the reference, creating the pumps of “sensor”, parameterizing them (Fig 9), and still ignoring the control rules for now;

9. Using Fig 11 as the reference, adjusting the inlets of delta pumps to the converted soil moisture storage (i.e. SoilBlock2’ in Fig 6);

10. Creating control rules for the emulator (Fig 9); and

11. Setting miscellaneous items in the model such as inflow, rainfall, time, etc. The SWMM model must use dynamic wave routing and the recommended flow unit system would be CMS.
